# Lack of association between breastfeeding duration and body mass index in children and adolescents – A Swedish cohort study

**DOI:** 10.1371/journal.pone.0319502

**Published:** 2025-03-12

**Authors:** Orsolya Haahr Vad, Lisa Önnestam, Kristina Bengtsson Boström, Åsa Jolesjö, Jenny Sandegård, Tobias Andersson

**Affiliations:** 1 Närhälsan Vårgårda Health Care Centre, Vårgårda, Sweden; 2 Närhälsan Nossebro Health Care Centre, Nossebro, Sweden; 3 Hamnstadens Health Care Centre, Lidköping, Sweden; 4 Regionhälsan R&D Centre, Skaraborg Primary Care, Skövde, Sweden; 5 School of Public Health and Community Medicine, Institute of Medicine, Sahlgrenska Academy, University of Gothenburg, Gothenburg, Sweden; 6 Närhälsan Ågårdsskogen Health Care Centre, Lidköping, Sweden; Bahir Dar University, ETHIOPIA

## Abstract

**Aims:**

The aim of the study was to investigate the association between breastfeeding duration and body mass index (BMI), overweight and obesity in children during follow-up until 16 years of age.

**Methods:**

Observational cohort study of mothers and their children born 1999–2000 in a municipality in southwestern Sweden. Data were retrieved from antenatal clinics, primary care child health care centres and school health care. The study exposure was breastfeeding duration measured in months and categorised by duration < 6 months and ≥ 6 months. The study outcomes were development of BMI, and overweight and obesity according to ISO-BMI.

**Results:**

The study population comprised 312 mothers and their 319 children of whom 120 were breastfed < 6 months and 199 ≥ 6 months. The overall partial or exclusive median breastfeeding duration was 6.5 months. No associations were found between breastfeeding duration in months and BMI in unadjusted (*p* = 0.70) and adjusted (*p* = 0.92) linear mixed-effects models with repeated BMI recordings at approximately 4, 7, 10, 13 and 16 years. Further, no associations were found in subgroup analyses for girls and boys. The adjusted analyses were adjusted for maternal age, smoking, BMI and parity, and for the child’s sex, gestational age at birth and birth weight. Unadjusted logistic mixed-effects models with repeated ISO-BMI classifications at approximately 4, 7, 10, 13 and 16 years showed no associations between breastfeeding duration (≥6 months versus < 6 months) and overweight or obesity as compared to underweight or normal weight, in total (odds ratio 1.46, 95% confidence interval 0.69–3.08) or in boys and girls separately.

**Conclusions:**

We found no association between breastfeeding duration and childhood and adolescence BMI up to 16 years of age or the development of overweight or obesity.

## Introduction

Overweight and obesity are major challenges to health, as they often lead to the development of chronic diseases [1]. Since the economic impact of these conditions is very high, prevention of overweight and obesity and their consequences during the life course is essential [2]. Childhood overweight and obesity are some of the most serious public health concerns of the 21st century [1], and children with overweight and obesity at preschool age also have an increased risk of obesity in later life [3]. In a child, obesity increases the risk of mental illness, psychological stigma and social exclusion [4]. Childhood obesity also increases type 2 diabetes morbidity and mortality and premature mortality from cardiovascular disease [5]. Treatment success of childhood obesity depends heavily on adherence to recommendations. Healthy eating and physical activity are basic requirements. Without adherence of child and family, interventions are not likely to be effective [6].

There has been an increased worldwide prevalence of childhood and adolescent obesity from 1975 to 2016. The age-standardised prevalence (5-19 years) increased in girls from 0.7% to 5.6% and from 0.9% to 7.8% in boys. The prevalence of obesity was > 30% in the Pacific Islands and > 20% in the Middle East, north Africa, the western Pacific as well as in the Caribbean and in the USA in 2016 [7]. In 2020, according to the Public Health Agency of Sweden, overweight and obesity were present in 21% of 16–19-year-old high school students [8]. The causes of overweight and obesity are both genetic and environmental [9]. A systematic review and meta-analysis showed that maternal body mass index (BMI), in particular, was associated with child obesity [10].

Breastfeeding is the optimal way of feeding infants with many beneficial effects, both for the child and for the nursing mother [11]. The positive health effects for the child associated with breastfeeding include positive hormonal and immunological effects and protection against gastrointestinal and respiratory tract infections [12]. Several studies have also shown an association between breastfeeding and reduced incidence of overweight and obesity in children [11,13–15]. Conversely, some studies did not find significant associations between breastfeeding and BMI in children [16,17]. One study that investigated the association between breastfeeding and BMI in a cohort of Australian children from birth to 21 years of age found no significant difference in overweight and obesity between the children breastfed for at least 6 months compared to the children who were not breastfed [18]. There are relatively few recent studies examining the relationship between breastfeeding duration and childhood and adolescent obesity with follow up until 16 years of age or longer [14].

The aim of this study was to investigate the association between breastfeeding duration recorded by child health care centres and overweight and obesity in children followed until 16 years of age in a cohort of mothers and children in a Swedish municipality.

## Materials and methods

In Sweden, child health care centres are organised in primary health care, where registered specialist paediatric or district nurses work alongside general practitioners. Both professions advise on breastfeeding [19] and record the length of breastfeeding in medical records. These records can be used for studies of breastfeeding in relation to different outcomes in children.

### Study population and data collection

The study population and data collection used in this observational cohort study have been described earlier [20]. In short, the study population included mothers and their children born 1999–2000 that were followed at child health care centres in a southwestern Swedish municipality with approximately 38 000 inhabitants in 1999. The municipality consisted of a small urban area surrounded by rural areas. Individual data were collected from primary care child health care centres using the unique personal identity number assigned to every Swedish resident [21]. The data obtained from the child health care centres were retrieved with start in 2000 and included sex, gestational age at birth, body weight and height at birth, body weight and height from 0–6 years and occurrence of breastfeeding of the child, which was recorded at each visit. Occurrence of breastfeeding included both exclusive and partial breastfeeding. Similar, the mothers’ body weight and height, BMI, age, parity and smoking habits were retrieved from the antenatal clinics. Data on weight and height at 7–16 years of age were retrieved between 25 March 2019 and 6 February 2020 using school health care records on routine measurements of body weight and height at approximately 7, 10, 13 and 16 years of age. If a child had multiple adjacent visits, data were chosen from the visit closest to 4 (mean age 4.1 ±  standard deviation 0.19 years), 7 (6.9 ± 0.53), 10 (10.4 ± 0.54), 13 (13.2 ± 0.76) and 16 (16.4 ± 0.58) years of age. As the BMI for children varies with age and sex, ISO-BMI was assessed with cutoffs for underweight, overweight and obesity according to Cole’s classification [22,23] at the exact age at the time of the examination.

### Statistical analysis

We used descriptive statistics for the baseline data. The breastfeeding duration of the children was categorised into two groups: < 6 months and ≥ 6 months, based on the World Health Organization’s recommendation of 6 months of exclusive breastfeeding [24]. Boxplots were used to describe the BMI of the girls and boys according to age and breastfeeding duration. To preserve individual confidentiality, potentially identifying outliers were not graphically presented but were included in the analyses. Unadjusted linear regression models were used to estimate the association between breastfeeding duration and the mother’s BMI and parity. Simple autoregressive linear regression models were used to estimate the association between breastfeeding duration in months and the children’s BMI as a continuous variable. In these models, adjustment was made for BMI at the age immediately preceding ages 7, 10, 13 and 16 years. To adjust for potential confounders, multiple autoregressive linear regression models were additionally adjusted for maternal age, parity, smoking and BMI, gestational age at birth, weight at birth and the child’s sex. In addition, we used linear mixed-effects regression models with repeated measurements at the exact ages at the examinations to estimate the association between breastfeeding duration (in months) and children’s BMI as a continuous variable. To address the correlation of repeated BMI measurements within individuals, the models included a random intercept for each child. Both unadjusted and multiple adjusted models were fitted. The adjusted models also accounted for potential confounders, including maternal age, parity, smoking and BMI, gestational age at birth, weight at birth and the child’s sex. We also fitted unadjusted and multiple adjusted linear mixed-effects models, including a three-way interaction term between breastfeeding duration, sex, and age, to predict and visualize BMI with 95% confidence intervals for boys and girls across different breastfeeding durations at specific ages.

The proportion of girls and boys with underweight or normal weight compared to overweight or obesity, based on ISO-BMI at approximately 4, 7, 10, 13 and 16 years of age, were presented graphically according to breastfeeding duration < 6 months and ≥ 6 months. Binary logistic autoregressive regression models were used to estimate the odds ratios (OR) for children to be classified as overweight/obese compared to underweight/normal weight according to ISO-BMI, by duration of breastfeeding (≥6 months versus < 6 months). In these models, we adjusted for the BMI at the age immediately preceding ages 7, 10, 13 and 16 years. In addition, we used logistic mixed-effects models with repeated ISO-BMI classifications at approximately 4, 7, 10, 13 and 16 years of age to estimate the OR for children to be classified as overweight/obese compared to underweight/normal weight according to ISO-BMI, by duration of breastfeeding (≥6 months versus < 6 months). With respect to the limited number of children with overweight/obesity in each age group, no further adjustments were made in the logistic regression models.

Statistical analyses were performed for all the children and separately for the girls and boys. As some mothers had multiple births, clustered standard errors were used in the regression models. All tests were two-tailed and conducted at a 0.05 significance level. The statistical analyses were performed using Stata 18.0 (Stata Corp., College Station, TX, US).

### Ethics

The study was approved by the Regional Ethics Review Board in Gothenburg (reference 935-18). The authors collecting data had access to information that could identify individual participants. Thereafter, the data were pseudonymised before analysis. Informed consent was not retrieved from the studied individuals since the results are reported from retrospective data from registers. The need for consent was waived by the Regional Ethics Review Board in Gothenburg.

## Results

**Fig 1:**
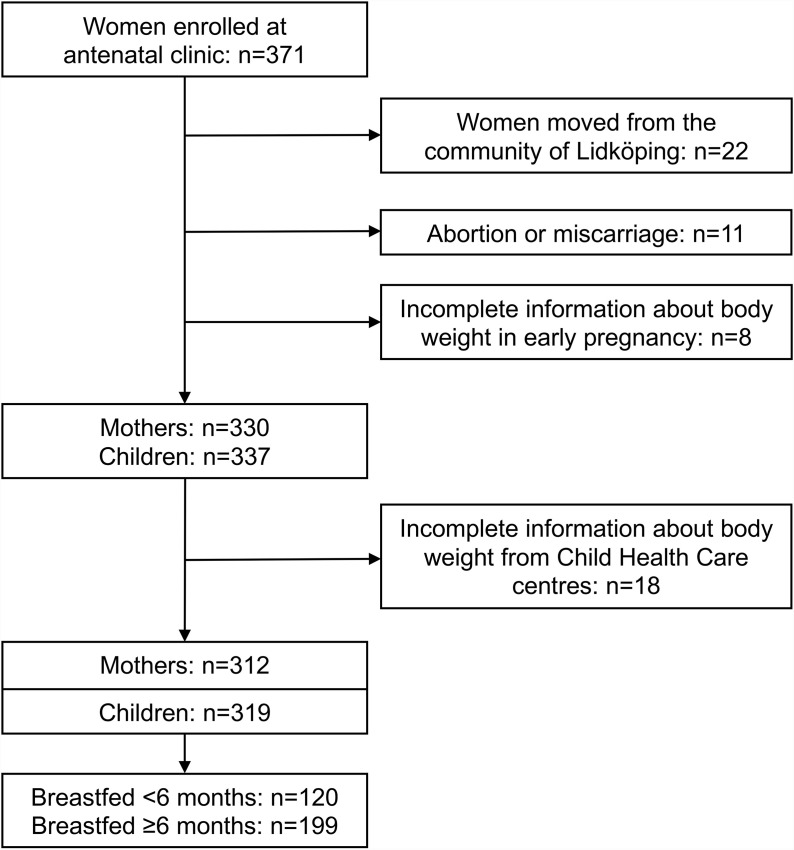
Flowchart of mothers and children included in the study.

The study population comprised 312 mothers and their 319 children (i.e., 319 mother–child dyads; see Fig 1). The median duration of breastfeeding was 6.5 months (interquartile range 4–8 months, total range 0–36 months). Neither the mothers’ BMI (*p* = 0.20) nor parity (*p* = 0.88) was associated with the length of breastfeeding.

Table 1 presents the maternal and child characteristics by duration of breastfeeding (<6 and ≥ 6 months). Of the 319 children (151 girls and 168 boys), 120 children were breastfed for less than 6 months, and 199 children were breastfed for 6 months or more. Most mothers were non-smokers. Parity varied between 0 and 7 (median 1; interquartile range 0–2).

**Table 1:** Maternal and child characteristics by breastfeeding duration in a cohort of 319 mother–child dyads in a community in southwest Sweden.

	Total	BF < 6 months	BF ≥ 6 months
Maternal characteristics, *n*	312	114	198
Age (years)	28.8 (5.0)	27.5 (5.6)	29.6 (4.4)
Parity	1.0 (0–2.0)	1.0 (0–2.0)	1.0 (0–2.0)
Smoking, yes	20 (6.4)	12 (10.5)	8 (4.0)
** **Height (cm)	166.4 (5.6)	165.7 (5.7)	166.8 (5.5)
** **Weight (kg)	68.5 (12.2)	68.9 (13.5)	68.3 (11.5)
** **BMI (kg/m^2^)	24.7 (4.1)	25.1 (4.5)	24.5 (3.8)
Child characteristics, n	319	120	199
** **Sex			
** **Girls	151 (47.3)	54 (45.0)	97 (48.7)
** **Boys	168 (52.7)	66 (55.0)	102 (51.3)
** **Birth weight (kg)	3.6 (0.6)	3.5 (0.7)	3.7 (0.5)
** **Girls	3.5 (0.6)	3.3 (0.8)	3.6 (0.5)
** **Boys	3.7 (0.6)	3.6 (0.6)	3.8 (0.5)
** **Birth week	38.8 (2.0)	38.4 (2.6)	39.0 (1.5)
** **Girls	38.6 (2.2)	38.0 (3.0)	38.9 (1.6)
** **Boys	39.0 (1.8)	38.7 (2.1)	39.1 (1.5)
** **BF duration (months)	6.5 (4.0–8.0)	2.8 (1.5–4.5)	8.0 (7.0–9.5)
** **Girls	7.0 (4.0–8.0)	2.5 (2.0–4.0)	8.0 (7.0–10.0)
** **Boys	6.0 (3.5–8.0)	3.0 (1.3–4.5)	8.0 (7.0–9.0)

The mean ( ± SD) is presented for the continuous variables except for parity and breastfeeding duration where the median (interquartile range) is presented. For the categorical variables, the n (%) is presented. BF, breastfeeding; BMI, body mass index.

**Fig 2:**
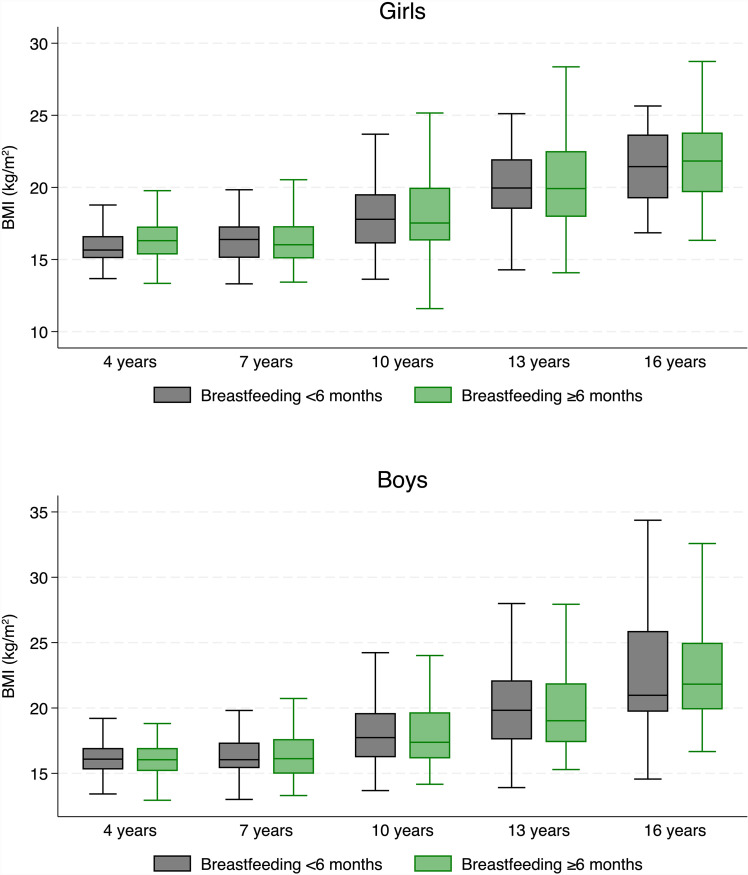
Boxplots of body mass index for girls and boys according to age and breastfeeding duration (<6 months versus ≥ 6 months). Outliers have been omitted due to confidentiality to not present individual data.

Fig 2 shows the boxplots of unadjusted BMI according to age group, sex and duration of breastfeeding (<6 and ≥ 6 months). BMI increased with age in both sexes, as expected. In the autoregressive simple linear regression models using continuous variables, no significant associations were found between breastfeeding duration and the children’s BMI for any ages between 4 and 16 years (see Table 2). In the multiple autoregressive linear regression models adjusted for maternal age, parity, smoking and BMI and for gestational age at birth and weight at birth, a significant association was seen for 10-year-old girls, but not for boys or any other ages (see Table 2).

**Table 2:** Association between breastfeeding duration in months and body mass index of children, estimated by autoregressive simple and multiple linear regression models.

	Unadjusted model			Adjusted model^a^		
	Coefficient	95% CI	*p*	Coefficient	95% CI	*p*
4 years						
** **All, n = 318	-0.003	-0.046 to 0.040	0.88	-0.011	-0.052 to 0.030	0.60
** **Girls, n = 151	0.005	-0.055 to 0.066	0.86	-0.009	-0.060 to 0.042	0.72
** **Boys, n = 167	-0.018	-0.082 to 0.045	0.57	-0.020	-0.097 to 0.057	0.61
7 years						
** **All, n = 313	-0.022	-0.067 to 0.022	0.33	-0.009	-0.051 to 0.034	0.70
** **Girls, n = 150	-0.026	-0.084 to 0.032	0.37	-0.017	-0.074 to 0.041	0.57
** **Boys, n = 163	-0.008	-0.070 to 0.054	0.80	0.008	-0.058 to 0.073	0.82
10 years						
** **All, n = 314	0.031	-0.010 to 0.072	0.14	0.049	0.003–0.094	0.035
** **Girls, n = 150	0.048	-0.002 to 0.098	0.059	0.066	0.010–0.122	0.022
** **Boys, n = 164	0.004	-0.070 to 0.078	0.91	0.014	-0.071 to 0.098	0.75
13 years						
** **All, n = 311	-0.030	-0.073 to 0.013	0.17	-0.033	-0.079 to 0.012	0.15
** **Girls, n = 148	-0.023	-0.080 to 0.035	0.43	-0.017	-0.074 to 0.039	0.55
** **Boys, n = 163	-0.041	-0.103 to 0.020	0.19	-0.054	-0.119 to 0.012	0.11
16 years						
** **All, n = 270	0.001	-0.063 to 0.066	0.97	0.011	-0.057 to 0.080	0.74
** **Girls, n = 129	0.003	-0.066 to 0.073	0.93	-0.0002	-0.069 to 0.068	0.99
** **Boys, n = 141	0.029	-0.099 to 0.156	0.66	0.026	-0.130 to 0.183	0.74

In all models, for the approximate ages 7, 10, 13 and 16 years, adjustments were made for body mass index at the immediately preceding age.

^a^Additionally adjusted for maternal age, parity, smoking and body mass index and for child’s sex, gestational age at birth and birth weight.

**Fig 3:**
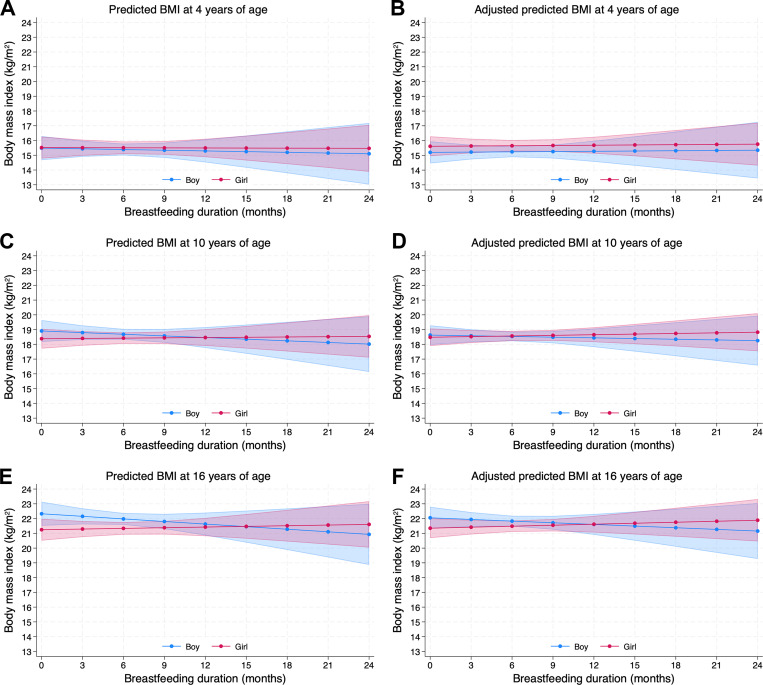
Predicted BMI by breastfeeding duration for boys and girls at ages 4, 10, and 16 years. Predicted BMI at age 4 years is shown in Panel A for the unadjusted model and in Panel B for the multiple adjusted model. Panels C and D present the corresponding predictions at age 10 years for the unadjusted and adjusted models, respectively. Similarly, predictions at age 16 years are displayed in Panels E (unadjusted) and F (adjusted). Across all ages, the 95% confidence intervals for BMI overlapped across different breastfeeding durations, indicating no statistically significant association between breastfeeding duration and BMI for either boys or girls.

In the unadjusted and adjusted linear mixed effect analyses, no associations were found between breastfeeding duration and BMI up to 16 years of age (*p* = 0.70 and *p* = 0.92, respectively; see Table 3). Further, in these analyses, no associations were found in the separate analyses for girls and boys. Predicted BMI by breastfeeding duration at ages 4, 10, and 16 years is presented in Fig 3, with wide 95% confidence intervals indicating no statistically significant association between breastfeeding duration and BMI for either boys or girls.

**Table 3:** Association between breastfeeding duration in months and body mass index of children up to 16 years of age, estimated by linear mixed-effects models with repeated measurements of body mass index at approximately 4, 7, 10, 13 and 16 years of age.

	Unadjusted model			Adjusted model^a^		
	Coefficient	95% CI	*p*	Coefficient	95% CI	*p*
All, n = 319	-0.013	-0.076 to 0.051	0.70	0.003	-0.056 to 0.061	0.92
Girls, n = 151	0.007	-0.062 to 0.076	0.85	0.009	-0.058 to 0.077	0.79
Boys, n = 168	-0.038	-0.153 to 0.078	0.52	-0.019	-0.120 to 0.082	0.72

In the unadjusted overall model, the variance for body mass index attributable to differences between children (random intercept variance) was 4.91 (95% CI 4.12–5.86), while the residual variance was 3.12 (95% CI 2.88–3.38). In the adjusted model, the random intercept variance was reduced to 3.49 (95% CI 2.90–4.20), with the residual variance remaining at 3.12 (95% CI 2.88–3.38).

^a^Adjusted for maternal age, parity, smoking and body mass index and for child’s sex, gestational age at birth and birth weight.

**Fig 4:**
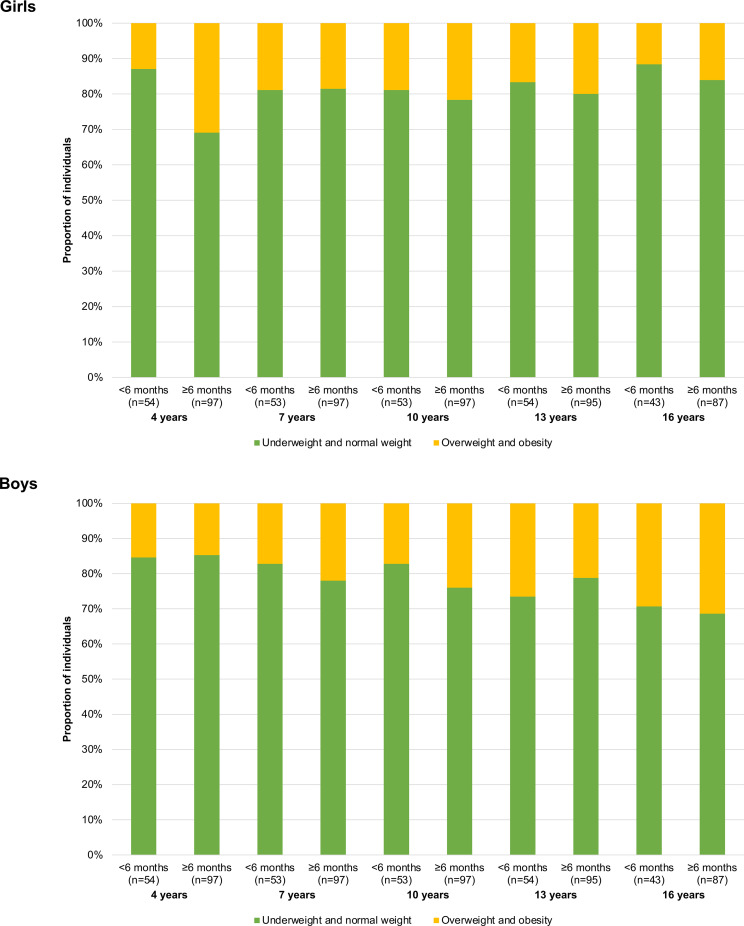
Proportions of girls and boys with underweight or normal weight compared to overweight or obesity according to ISO body mass index at different ages and by breastfeeding duration (<6 months versus ≥ 6 months).

Fig 4 presents the proportions of girls and boys with underweight or normal weight compared to overweight or obesity according to ISO-BMI at different ages and by breastfeeding duration (<6 months versus ≥ 6 months). Visually, the proportion of overweight or obesity increased with age in the boys. Among the 4-year-old girls, the proportion of overweight or obesity was visually larger among the girls who had been breastfed ≥ 6 months compared to < 6 months (30.9% versus 13.0%). However, this difference did not persist at older ages.

In the autoregressive binary logistic regression models, there were no significant associations with breastfeeding duration ≥ 6 months compared to < 6 months overall for children to be overweight/obese compared to underweight/normal weight (see Table 4). The subgroup analyses showed inconsistent results with wide 95% confidence intervals and alternating directions of the OR, with significant effects only for the 4-year-old girls and 13-year-old boys. The logistic mixed-effects models with repeated ISO-BMI classifications at approximately 4, 7, 10, 13 and 16 years of age showed no significant associations between overweight/obesity compared to underweight/normal weight and duration of breastfeeding (≥6 months versus < 6 months), in total (OR 1.46, 95% CI 0.69–3.08), for the girls (OR 1.70, 95% CI 0.58–4.96) or for the boys (OR 1.35, 95% CI 0.46–4.01).

**Table 4:** Odds ratios for children to be classified as overweight/obese compared to underweight/normal weight according to ISO-BMI, by duration of breastfeeding (≥6 months versus < 6 months).

	Odds ratio	95% CI	*p*
4 years			
** **All, n = 318	1.75	0.95–3.22	0.071
** **Girls, n = 151	3.01	1.21–7.44	0.017
** **Boys, n = 167	0.95	0.40–2.25	0.90
7 years			
** **All, n = 313	0.79	0.40–1.57	0.50
** **Girls, n = 150	0.33	0.09–1.14	0.079
** **Boys, n = 163	1.66	0.66–4.15	0.28
10 years			
** **All, n = 314	1.42	0.66–3.04	0.37
** **Girls, n = 150	1.31	0.46–3.70	0.62
** **Boys, n = 164	1.51	0.49–4.65	0.48
13 years			
** **All, n = 311	0.60	0.28–1.27	0.18
** **Girls, n = 148	1.21	0.36–4.09	0.76
** **Boys, n = 163	0.33	0.12–0.94	0.039
16 years			
** **All, n = 270	1.05	0.51–2.19	0.89
** **Girls, n = 129	0.96	0.28–3.32	0.95
** **Boys, n = 141	1.30	0.50–3.38	0.59

The odds ratios were estimated by autoregressive binary logistic regression models where, for the approximate ages 7, 10, 13 and 16 years, adjustment was made for body mass index at the immediately preceding age.

## Discussion

This Swedish observational study found that the duration of breastfeeding in a cohort of children born in 1999–2000 was not associated with BMI or ISO-BMI in girls and boys followed up to 16 years of age. The proportion of overweight or obesity was larger among the girls breastfed ≥ 6 months compared to < 6 months at the age of 4 years. However, this difference did not persist at older ages, and no statistically significant differences were found between the boys and the girls.

### Findings in relation to other studies

National breastfeeding data from 1999–2000 from the Swedish National Board of Health and Welfare show a breastfeeding rate of 83% for more than four months and 72% for more than six months [25]. This frequency is higher than in our study, in which 62% of the children were breastfed for more than six months, which could be explained by socioeconomic factors that have been shown to influence, among other factors, breastfeeding frequency [26]. Our results are in line with results from some other Scandinavian studies from the same period that found no significant association between breastfeeding and child weight. A Swedish prospective cohort study found that the duration of exclusive breastfeeding was not associated with obesity in early childhood after adjusting for confounding factors [27]. Another study based on 7‐year‐old and 11‐year‐old children born 1997–2003 enrolled in the Danish National Birth Cohort found that a longer duration of exclusive breastfeeding did not seem to protect 5-month-old infants with high weight for age from overweight in childhood [28]. In addition, the Belarusian cluster-randomised Promotion of Breastfeeding Intervention Trial (PROBIT), which allocated maternity hospitals into intervention or control arms in 1996–1997, showed no significant effect of breastfeeding on childhood obesity and even a negative association regarding overweight [29]. In contrast, a recent systematic review and meta-analysis that included 159 studies of children born from 1934 to 2017 showed that breastfeeding was associated with a significantly reduced risk of overweight and obesity [14]. Another meta-analysis showed a dose-response protective effect of breastfeeding duration on childhood obesity [30].

Even though our study did not show an association between breastfeeding duration and overweight and obesity, breastfeeding remains important for its numerous health benefits for both mother and child [11,12]. It may be important to recognize the possibility that there might be no direct or strong link between breastfeeding duration and a reduced risk of obesity in children as breastfeeding is not an option for all women due to health issues or personal reasons. Health care interventions should be grounded in a broader understanding of the many contributing factors to childhood obesity and not just one single factor, such as breastfeeding. Obesity is influenced by a multitude of factors, including genetics, diet, physical activity, socioeconomic status, and environmental factors [1].

Another possible consideration related to the null finding of our study is the composition of modern infant formula, which has become increasingly similar to breast milk in terms of nutritional content [31,32]. This similarity may reduce any potential protective effect that breastfeeding could have against obesity, as infants who are formula-fed may receive comparable levels of essential nutrients. Therefore, differences in BMI or obesity risk between breastfed and formula-fed children may be diluted, particularly when other factors influencing weight gain are accounted for.

### Strengths and limitations

In Sweden, children are offered free health care at child health care centres in primary health care. About 99% of children in Sweden aged 0–6 years participate in the programme. Thereafter, school health care with specialised nurses offers regular health visits up to the 12th grade [33]. Accordingly, one strength of our study was that most of the children born 1999–2000 were included in this study and were continuously monitored in a real-world setting over a long-term period. Also, data on breastfeeding were prospectively recorded, especially frequently during the first 6 months, reducing the risk of recall bias, which could be the case in retrospectively reported data. The data on height, weight and smoking were retrieved from records from antenatal clinics and child health care centres in primary health care and from school health care data that are also used in national registers [25]. However, this study also had several limitations, including incomplete data on the addition or composition of milk formula. Further, we had no data on frequency of exclusive or partial breastfeeding which could possibly influence the results. The study also had a small sample size with risk of underpowered analyses and no data on socioeconomic status, which might be important for the frequency and duration of breastfeeding. Likewise, data on factors that can influence BMI development, such as lifestyle, psychological and physical illnesses in the children, were not available. Even though we found no association between breastfeeding duration and BMI when using continuous data, the dichotomization of breastfeeding duration into < 6 months and ≥ 6 months poses a limitation. Specifically, the < 6 months category is heterogeneous, including mothers who breastfed for only a short period alongside those who breastfed for nearly six months, which may obscure nuanced effects on BMI. In addition, although the study is based on a real setting in child health care centres and many other countries have similar conditions with regular health check-ups for children, the frequency and duration of breastfeeding may differ among populations and our study might not be relevant in countries with different breastfeeding patterns.

## Conclusion

We did not, contrary to most other studies, find any significant associations linking breastfeeding duration with child and adolescent BMI up to 16 years of age or the development of overweight or obesity according to ISO-BMI. Further research is needed to better understand the complex interactions between infant feeding practices and long-term health outcomes.
